# Editorial Department

**Published:** 1885-07

**Authors:** 


### The Bistoury.

ELMIRA, N. Y., JULY, 1885.

The BISTOURY is published Quarterly, upon the first of April, July, October and January, at Fifty Cents a year in advance.

THE BISTOURY may be found on file at Geo. P. Rowell & Co's Newspaper Advertising Bureau, 10 Spruce Street, New York.

### Editorial Department.

IN THE eye and ear clinic of the Surgical Institute we are in special force, having not only the advantage of years of experience, but also the special study and practice in latest discoveries of our son and partner, Thad S. Up de Graff, jr., M. D.

---

A TYPOGRAPHICAL ERROR.—We find in reading our proof among other ingredients of a prescription—powdered tail. It should have read—powdered talc.

It would, no doubt, be a puzzler to the druggist to fill the above. They are very ambitious, however; so no doubt the article could be found.

---

SAYS the Albany *Argus:* "The "Bung" "party, as the beer interest is called in Eng- "land, is giving poor Mr. Gladstone more con- "cern than Russia and the Mahdi combined. It "is all powerful at the election." And now, while we are reading, the news comes that Gladstone has fallen and gone out of power before the "Bung" party. But still men keep saying, don't, don't organize for prohibition ! The "Bung" party is in power. What are we going to do about it ?

---

RINK HYGIENE.—The plodding away under canvas around the track of a roller rink, inhaling the vitiated air expelled from many lungs and saturated rendered offensive by the numerous perspiring bodies in the room, is far from enervating. The fuel gas is poisonous to the system. If a person attempts to breathe a less proportion of oxygen than what is normally contained in the air, the effect is depressing to all the vital functions, physical and mental, to an extent corresponding to the deficiency.

If parents wish to have their children attain a strong physique, they should let them run in the open air more.

OUR FRIEND, T. K. Beecher, says that the money question is not settled yet. That there are but five questions worth discussing—liquor, labor, money, lands, and property. These, he says, are his questions, and so the leaders of the two parties fight shy of them. Mr. Beecher has a notion that gold and silver are metals and ought to be sold by weight and not by count, just as copper is.

---

TEMPERANCE.—Some of our friends in the larger cities where the front doors of the saloons are closed by law on Sundays and the back doors kept open, tell us they propose to petition for the opening of the saloons entirely, as on that day now, there are more drunkards on the street than on weeks days.

Perhaps, high licenses, *almost* to prohibition, might remedy some of the evils from rum. Total prohibition has been ineffective in almost all places where tried.

---

UTILITY, beauty, economy, and safety demand that telephone and telegraph and electric light wires should come down and go underground. Before many years the antics of lightning playing over our vast webs of wire will convert all hands to our opinion. Possibly, too, men may yet discover that this elemental fire like the fabled chariot of the sun is too mettlesome to be safely driven by any Phæton, however ambitious, ingenious, and scientific.

Much as we are using electric forces, little, as yet, is understood as to their origin and essence.

---

THE BABY SPEAKS.—Well! I came out of the cataract alive, and that's more than I expected. I was then rubbed till I thought my skin was on fire. And then the strangest thing happened. I had already been led to expect many curious and startling things, but that was so ridiculous that I absolutely laughed. I do not think that that stupid nurse of mine detected my laugh, but I felt it bubbling within me all the time certainly. Things were brought to me in a pretty basket; they took one article and fastened it around my body, then another which they passed over my head, forcing my arms through two holes, then another and another, and finally one so long that I lost my other end. Then they put each foot of mine in a little bag, after which they told me to stand up like a man and go and see my mother.—*Babyhood.*

THE TENNIS CLUB.—The Elmira Tennis Club has fitted its grounds in admirable shape. The new pavilion is very attractive, it certainly is a great convenience to the members, and is admired greatly by their friends.

The club contains some of the prettiest and most expert lady players in the state.

Tennis is certainly the most invigorating amusement for both sexes that we possess, and ought to be encouraged.

---

GREAT BRITAIN.—Our neighbors seem to have had their share of troubles both internal and external of late and even Gladstone has at last failed to retain his supremacy.

The mills of the gods work slowly but surely; out of the throes of kingdoms may come better and happier governments for the people. At all events the mills seem to be very busy and we can only watch and await results.

---

THE WAR.—The magazines and weekly journals are fighting over the battles again, on paper this time, but the Generals on both sides are telling their stories and be it said they are exceedingly interesting reading and occasionally drop some information of the ways and means in use at Head Quarters in those days, which do not shock us as much now as it would have then.

Anecdotes of Lincoln and his cabinet are also being published which have been heretofore confined to the knowledge of a few. Let us have it all. It can do no harm now.

---

A WELL-KNOWN Elmira clergyman to illustrate the *cram* that passes for education in many girls' schools, vouches for the following from an examination" : *Teacher:* Did Martin Luther die a natural death? *Fair girl with flowers:* No. He was ex-communicated by a Pope's bull.

The pastor would do well to add to his illustrations from *Texas Siftings*, in these days when physiology, hygiene, and narcoties are studied at school. *Teacher:* "So you can't do a simple sum in arithmetic. Now, let me explain it to you. Suppose eight of you have together forty-eight apples, thirty-two peaches, and sixteen melons, what will each one of you get?"

"Colerer morgus," replied Johnny Fizzletop, who is addicted to that malady.

---

ADVERTISING.—What a wonderful art it has become, and especially noticeable is the ingenuity of the theatrical profession in getting themselves freely advertised. Lost jewels, divorces, and other well known dodges have ceased to be effective. The papers are advertising Mdlle. Nevada through the medium of a warfare between Herr Swab, an occasional critic for some of the New York papers, and herself, with a question of blackmail in dispute. However the difficulty, if there is any, may be settled, the advertising is good and THE BISTOURY does not mind giving it a boost.

---

SEA-SICKNESS.—Reader have you ever been sea-sick? Well don't! We have made a voyage, since the last issue of THE BISTOURY, to Bermuda. Now whether the sea between New York and Bermuda possesses any special privileges or advantages in the way of producing that malady over any other portion of the ocean we are not informed, nor do we want to be. We do know, however, just exactly what liberty it will take with a fellow when it gets him down on that "Sea in Ships."

We are not going to attempt to describe the disease or offer any preventives or cures for it, except to advise you not to try it. It is the quintessence of all mean sickness, and combines in its miserable symptoms every disagreeable pain, ache and misery that can be conceived.

An excellent way to avoid it is to stay ashore.

---

THE NEXT QUESTION.—Before many years the question will arise in many homes *what to do with the ashes of our dead*—four or five pounds of pearl-grey purity that has come from the crematory.

We suggest that the sorrowing friend should *wait* and let the question settle itself in one way in one family, in another way in another. There should be no *fashion* in this matter—no rule about it. The ashes may rest in Woodlawn while a monument marks the spot. They may be inurned and set in some sacred spot of home. They may be treasured in the bureau drawer side by side with locks of hair or the little shoes of the baby last gone from the world of the dying. They might repose underneath the altar of our churches innocuous. They might go to our flower garden and from them let violets spring. They might repose at the roots of a memorial tree or a clinging ivy vine. Just wait, and the heart will settle the question what to do. Wait and do the thing that brings peace.

STATE DAM.—Our friend Chandler has just returned from trout fishing at State Dam, near Malone, and reports great improvements there, especially in the corduroy road at the last of the journey. This, under the supervision and expenditure of Mr. Lowe, the landlord, has been changed into a decent summer road. The trout are as abundant as ever and as ready to be taken. The internal improvements in the house add to the comfort of the guests and the table never has needed any improvement.

The flies and mosquitoes are no worse there than in any other part of the Adirondacks, and Mr. Chandler has proven the following recipe taken from "The Angler" to be an infallible preventive as well as a cure for the bites:

Three parts olive oil.
Two parts oil of pennyroyal.
One part glycerin.
One part ammonia.

To be well shaken before applying to face and hands. Avoid getting the mixture into the eyes. Armed with this the trout fisherman can have glorious sport at State Dam any time during the season.

---

DIAGNOSIS OF DISCONTENT.—Only a few years ago Jay Gould was earning two dollars a day as a surveyor in Western New York. He came to the city, and by "operations" not generally understood, gained control of millions.

A mechanic in the little village where Gould started is still earning by hard work the same old $2.50 per day or less, and barely makes both ends meet by practicing extreme frugality. He has known Gould and fails to note any deserving in him surpassing that of other men all around him. As he broods the matter, he feels that there must have been some crookedness or knavery in the process of swift accumulation. He ceases to respect the right of Jay Gould to his alleged property. Having come so far, the step is a short one to the disregard of all property rights—that is, to communism pure and simple. And now all honest men begin to suffer, reasonably fearing that the safety of their little properties thriftily and honestly acquired, and communism becomes the bug-a-boo of the day.

Senator Platt, of Connecticut, puts these ideas in form as above, and gives the *rationale* of the alarming spread of communistic sentiment all over the land. "Operators" in money and stocks from highest to lowest are the cause of the discontents that seem to threaten both life and property.

---

LITTLE JOE.—Amidst the daily chronicles of crime and the prominence of the worst features of human nature, it is refreshing occasionally to find cropping out in least expected quarters the true ore of the human heart.

"Little Joe" was a waif in the streets of New York two years ago; he took to selling papers, went through all the kicking, cuffing, and abuse from the larger gamins, but steadily, from acts of kindness within his limited reach, gained the affections of his wild companions Poor Little Joe succumbed at last to the fatigue and exposure of his pursuit. The newsboys, one hundred in number, met in front of City Hall to express their feelings on the loss. They passed the following resolution:

*Resolved*—"That we all liked Little Joe, who "was the best newsboy in New York. Every-"body is sorry he died."

A collection was then taken up to send delegates to the funeral. He was buried Sunday June 7th. On the coffin was a plate purchased by the boys. This was the inscription:

LITTLE JOE,
Aged 14,
The best newsboy in New York,
WE ALL LIKED HIM.

There were no services, but each boy sent a flower to be placed on the coffin.

Think of the little hero of fourteen who could wring such a tribute as that from the gamins of New York!

---

BERMUDA AND ITS RESULTS.—We had intended to have given the readers of the BISTOURY in this number an extended account of what we saw in our trip to the Island, but we have been so busy looking after its results since our return we have had but little time for the pen. One of the gratifying results we can not resist noticing to our many friends. We found at the head of the Institute upon our return, enjoying and deserving the confidence of the patients, our son and namesake, Dr. Thad S. Up de Graff, jr., whose graduation from Jefferson College two years ago we took occasion to note and whose studies since in the special branches have eminently fitted him for the treatment of eye, ear and throat troubles.

We have admitted him to a partnership in the business and can now enjoy a rest when needed, with perfect confidence that all is being done and well done.

Our readers have for many years entered into our griefs and pleasures as set forth in the light editorials of the BISTOURY and we may be pardoned at this time for a little display of parental pride.

MALARIA.—There are slurs constantly thrown upon the medical profession because of the reference to the source of many diseases in the term at the head of this.

It is used as a general expression and not bound by arbitrary rules of medical definitions and used not unadvisedly when it refers to the effects of foul exhalations from any source, vitiated drinking water or badly ventilated houses and rooms.

Too much public reference to these matters cannot be made; population is becoming dense and every human being has the necessity in life of vitiating and destroying much of the vitality of surrounding atmosphere. Too much care then cannot be taken in avoiding unnecessary exposure to malaria influences.

CICADA.—The seventeen year Locusts are in season now and have come in on time.

The life of this insect is a curious one. Let us start with an adult just seventeen years ago. They resemble the harvest fly more than any other insect but differ in color; the eye is red and the prominent lines on the wings are red, the body is black. Just seventeen years ago there crawled out of a hole in the ground a curious looking creature without wings, which had, in its water proof lined tunnel in the earth, been lying for a short time awaiting a favorable sunny warm day in June to crawl out and follow its destiny. It came out enclosed in a semi-transparent yellowish coat fitted closely over the head, limbs and body, crawled up the trunk of an oak, basked in the sun until evening and then in the early hours of the night split open its coat in the back and crawled out and soon developed its embryo wings; during its brief life it, with a singular saw apparatus which is attached to its body, cut slits in the bark of some twigs and laid eggs for another generation. These eggs shortly developed stumpy youngsters who provided with tough heads for boring purposes fell to the ground and immediately burrowed down to the succulent roots of the tree. Here they fastened themselves and sucked away for seventeen years, until suddenly awakened by natural instincts, they started upwards on the same journey their ancestor had made seventeen years before and are now busy, starting other families for a seventeen year suck! Truly the works of nature are wonderful.

THE TRICYLE.—Rev. Thos. K. Beecher has lately purchased a Columbia tricycle. He pronounces it the finest machine that he has seen.

Mr. Beecher has gained in health considerably, and is loud in praise of the tricycle or bicycle as a health invigorator.

BLAKE.—Did you know Blake? Fine handsome fellow; one of those never to be passed unnoticed. His appearence always commanded respect and admiration and fully established his claim to the highest position among his race. By the few who were admitted to the privilege of his friendship he was held in the highest esteem. Ladies kissed him and he was the pet of all male friends. To our friend Hamlin he was nearest and dearest, a constant companion night and day. Blake had one enemy, a base one, a sneak and a poisoner. If there is justice beyond the graves of his race "a ministering angel shall *poor Blake* be whilst *his enemy* lies howling." Blake was the handsomest and finest Gordon setter we ever saw.

W. R. Warner & Co. have received the first premium at the World's Exposition, New Orleans, for great uniformity and solubility of their sugar-coated Pills. This is the 9th, World's Fair prize which attests to their excellence.

